# DiffRIS: Enhancing referring remote sensing image segmentation with pre-trained text-to-image diffusion models

**DOI:** 10.1016/j.fmre.2025.11.017

**Published:** 2025-12-27

**Authors:** Zhe Dong, Yu-Zhe Sun, Tian-Zhu Liu, Yan-Feng Gu

**Affiliations:** School of Electronics and Information Engineering, Harbin Institute of Technology, Harbin 150001, China

**Keywords:** Diffusion models, Referring remote sensing image segmentation (RRSIS), Cross-modal reasoning, Natural language processing (NLP), Aerial imagery

## Abstract

Referring remote sensing image segmentation (RRSIS) enables the precise delineation of regions within remote sensing imagery through natural language descriptions, serving critical applications in disaster response, urban development, and environmental monitoring. Despite recent advances, current approaches face significant challenges in processing aerial imagery due to complex object characteristics including scale variations, diverse orientations, and semantic ambiguities inherent to the overhead perspective. To address these limitations, we propose DiffRIS, a novel framework that harnesses the semantic understanding capabilities of pre-trained text-to-image diffusion models for enhanced cross-modal alignment in RRSIS tasks. Our framework introduces two key innovations: a context perception adapter (CP-adapter) that dynamically refines linguistic features through global context modeling and object-aware reasoning, and a progressive cross-modal reasoning decoder (PCMRD) that iteratively aligns textual descriptions with visual regions for precise segmentation. The CP-adapter bridges the domain gap between general vision-language understanding and remote sensing applications, while PCMRD enables fine-grained semantic alignment through multi-scale feature interaction. Comprehensive experiments on three benchmark datasets—RRSIS-D, RefSegRS, and RISBench—demonstrate that DiffRIS consistently outperforms existing methods across all standard metrics, establishing a new state-of-the-art for RRSIS tasks. The significant performance improvements validate the effectiveness of leveraging pre-trained diffusion models for remote sensing applications through our proposed adaptive framework.

## Graphical abstract

**Figure:**
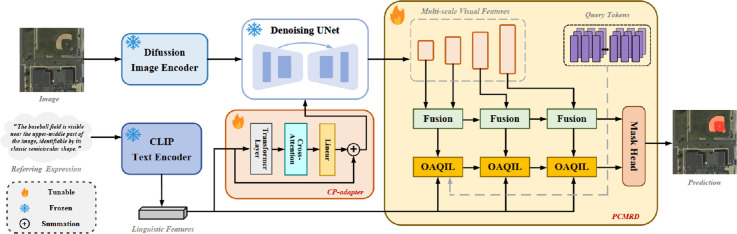

## 1 Introduction

Referring remote sensing image segmentation (RRSIS) aims to identify specific regions in remote sensing imagery based on given textual conditions, making it particularly suitable for practical applications such as defense reconnaissance [1], climate impact studies [2], urban infrastructure management [3], and land use categorization [4]. Unlike traditional single-modal segmentation methods [5,6], RRSIS leverages textual descriptions to guide image segmentation, overcoming the limitations of fixed category labels and enabling the processing of more diverse vocabulary and syntactic variations. However, the spatial and geographical disparities conveyed from an aerial perspective are fundamentally distinct from those in natural images, presenting significant challenges in achieving accurate and contextually relevant visual segmentation. Therefore, effectively harnessing textual conditions to enhance segmentation precision and semantic consistency remains a critical challenge in RRSIS research.

In recent years, multimodal fusion methods have made significant strides in modeling the semantic relationships between images and natural language for RRSIS tasks. Various approaches have been developed to enhance the complementarity of visual and linguistic information, including attention mechanisms [[7], [8], [9]], multi-level feature fusion [10,11], auto-regressive vertex generation [12,13], and expression queries [[14], [15], [16]]. Attention mechanisms effectively establish correspondences between visual and textual data, facilitating more accurate semantic fusion and improving segmentation performance by associating image pixels or regions with relevant words in the text. Multi-level feature fusion methods integrate image features and textual information across multiple scales, enhancing the model’s sensitivity to small targets and intricate details. While these methods introduce additional computational overhead through multi-scale processing and feature aggregation, they can achieve more favorable accuracy-efficiency trade-offs by enabling selective refinement at appropriate scales rather than uniformly processing high-resolution features throughout the network. This strategic allocation of computational resources proves particularly beneficial when handling the large-scale, high-resolution imagery characteristic of remote sensing applications. Auto-regressive vertex generation empowers the decoder to directly produce coordinate sequences in the coordinate space, thereby mitigating encoding redundancy and uncertainty, while leveraging sequential information to guide the order and positioning of vertices for more accurate and complete segmentation. Lastly, expression query methods optimize segmentation by retrieving target regions based on varied queries, each reflecting a unique interpretation of the expression, which further refines target differentiation and recognition accuracy. However, despite these advancements, existing methods face notable limitations, including challenges in generalization to diverse and complex datasets, inefficiencies in handling ambiguously defined regions, and reliance on task-specific designs or predefined queries, which restrict their adaptability and robustness in addressing the inherent variability and complexity of RRSIS tasks.

Text-to-image diffusion models [17,18] have recently demonstrated remarkable capabilities in the field of generative modeling, particularly excelling in capturing intricate semantics and fine-grained visual details. By leveraging pre-training on large-scale image-text datasets (e.g., LAION-5B [19]), these models effectively learn and integrate both high-level semantic concepts and low-level visual attributes, enabling highly controlled generation through customizable textual prompts. Their exceptional scalability allows for the production of high-quality images characterized by rich textures, diverse content, and coherent structures, while also exhibiting flexibility in semantic composition and editing. Moreover, the latent visual features learned by text-to-image diffusion models show a strong correlation with the corresponding words in textual prompts. This implicit alignment capability further enhances their performance in cross-modal semantic understanding and generation, making them a powerful tool for multimodal applications.

Building on the aforementioned advantages, the integration of text-to-image diffusion models into RRSIS tasks holds significant promise. On the one hand, text-to-image diffusion models, through extensive pre-training on vision-language datasets, effectively capture the intricate semantic associations between natural language descriptions and target regions within remote sensing imagery. By achieving deep cross-modal alignment, diffusion models enable a more precise mapping of textual descriptions to corresponding regions in remote sensing images, thereby offering robust guidance for referring image segmentation in complex and heterogeneous scenarios. On the other hand, the latent feature representations generated by diffusion models encapsulate not only rich visual details but also comprehensive global contextual information, providing essential semantic support for identifying multi-scale and fine-grained targets within remote sensing imagery. Given the variability in target scales, ranging from minute structures to vast expanses, diffusion models excel by unifying low-level and high-level visual concepts during generation. This unified modeling approach significantly enhances the sensitivity to multi-scale targets, thereby addressing the unique challenges posed by remote sensing data.

To this end, we explore the application of text-to-image diffusion models in remote sensing scenarios and devise DiffRIS, an effective diffusion model tailored for referring remote sensing image segmentation (RRSIS). By leveraging the powerful pre-trained knowledge embedded in text-to-image diffusion models, DiffRIS bridges the gap between natural language descriptions and remote sensing images, enabling precise segmentation of complex and diverse regions. Besides, a context-perception adapter (CP-adapter) and a progressive cross-modal reasoning decoder (PCMRD) are incorporated into the proposed framework, with both designed to enhance cross-modal semantic alignment and improve fine-grained segmentation accuracy. Extensive evaluations on multiple benchmark datasets demonstrate that DiffRIS outperforms existing methods, setting a new benchmark for RRSIS tasks.

In summary, the main contributions of this study are summarized as follows.

1) We pioneer the first diffusion model-based framework for RRSIS tasks. By leveraging the rich, pre-trained knowledge embedded in text-to-image diffusion models, DiffRIS significantly improves cross-modal semantic alignment and boosts segmentation accuracy.
2) Two complementary modules are designed: the context-perception adapter (CP-adapter), which refines textual features by capturing contextual dependencies, and the progressive cross-modal reasoning decoder (PCMRD), which iteratively refines semantic alignment to ensure precise segmentation.
3) Extensive evaluations across three benchmark datasets demonstrate that DiffRIS achieves state-of-the-art performance, surpassing existing methods and establishing a new standard for RRSIS tasks in terms of both precision and robustness.

The remainder of the paper is organized as follows: Section 2 reviews related works on RRSIS. In Section 3, we describe the proposed methodology in detail. Section 4 presents a comprehensive set of experiments and in-depth analyses. Finally, Section 6 concludes the paper and offers insights into potential future research directions.

## 2 Related work

### 2.1 Diffusion models in remote sensing

The diffusion models, also referred to as the diffusion probabilistic models, have emerged as a powerful family of deep generative models. Essentially, it consists of a set of probabilistic generative models that systematically degrade data by injecting noise, subsequently learning to reverse this process to generate samples. Recently, diffusion models have spurred significant advancements in the field of remote sensing research, demonstrating a sustained and substantial impact.

Diffusion models have the capacity to synthesize realistic remote sensing images from existing images or given textual descriptions, thereby facilitating the advancement of various remote sensing applications. Ou et al. [20] initially employed a vision-language pre-training model to generate captions for remote sensing images, thereby obtaining preliminary text prompts. Subsequently, they utilized these text prompts to enable the Stable Diffusion [21] model to synthesize the desired remote sensing images. Geolocation and sampling time were employed as prompts in the Stable Diffusion model by Khanna et al. [22], effectively enhancing its ability to generate high-quality satellite images. Moreover, by utilizing relevant features of remote sensing images as control conditions, Tang et al. [23] refined the generative process of the Stable Diffusion model. Recent studies have focused on employing masks such as class labels [24], maps [25], and semantic layouts [[26], [27], [28]] as guiding images for diffusion models to achieve image-to-image generation.

Another application of diffusion models in the field of remote sensing involves image enhancement, such as super-resolution, cloud removal, and denoising. Shi et al. [29] utilized cascaded images of multispectral and hyperspectral data as conditional inputs for the diffusion model, leveraging valuable information captured from both modalities to generate high spatial-resolution hyperspectral images (HSIs). By employing text prompts and edge information [30] as guiding conditions, Czerkawski et al. [31] integrated cloud masks and diffuse cloud-free images to achieve cloud removal. Yu et al. [32] enhanced the diffusion model’s ability to mitigate system noise by simulating adverse imaging conditions for remote sensing satellites through the introduction of various attack disturbances in the input images.

### 2.2 Referring image segmentation

Referring image segmentation (RIS) is a complex multimodal task that necessitates effective coordination between language and vision for accurate target region segmentation, surpassing the challenges posed by visual question answering [33] and visual dialogue [34].

Early approaches utilized long short-term memory (LSTM) [35] for encoding linguistic representations while employing convolutional neural network (CNN) [36] to extract spatial features from images at multiple levels. The recursive multimodal interaction model (RMI) [37] employed a multimodal LSTM network for linguistic representation, integrating visual features and capturing spatial variations of multimodal information to generate coarse localization masks, which were subsequently refined through a unidirectional LSTM network. Hu et al. [38] achieved referring image segmentation by employing a CNN-LSTM framework to extract visual features from images and linguistic features from natural language representations. A recurrent refinement network (RRN) [39] was proposed to address the lack of multi-scale semantics in image representation, utilizing a feature pyramid to match each word with every pixel in the image to generate an initial segmentation mask, which is subsequently refined and iteratively optimized through a recursive optimization module.

Recent developments have increasingly favored the adoption of Transformers to enhance the feature extraction and integration of visual-language modalities. The language-aware visual Transformer (LAVT) [40] employed the Swin Transformer [41] as the visual backbone, integrating vision-language fusion modules within the final layers of the visual encoder. Ding et al. developed a vision-language Transformer (VLT) [42] framework to facilitate deep interactions among multimodal information, thereby enhancing the comprehensive understanding of vision-language features. CRIS [43] and ReSTR [44] employed analogous methodologies, utilizing dual transformer encoders for the preliminary encoding of modalities, which was subsequently succeeded by feature integration via a multi-modal Transformer encoder or decoder. Similarly, PolyFormer [13] and SeqTr [12] utilized a multi-modal Transformer for vision-language fusion, producing masks as sequences of contour points. In contrast, CGFormer [45] and GRES [13] treated Transformer queries as region proposals, framing RIS as proposal-level classification tasks.

### 2.3 Referring remote sensing image segmentation

Referring Remote Sensing Image Segmentation (RRSIS) is an emerging cross-modal task that enables precise localization and segmentation of target objects in remote sensing imagery based on natural language expressions. Unlike traditional semantic or instance segmentation, RRSIS must simultaneously address the intrinsic complexity of remote sensing data—characterized by diverse object scales, cluttered backgrounds, and intricate spatial relationships—and the semantic alignment gap between textual descriptions and high-resolution visual features. These challenges have driven rapid progress in approaches emphasizing cross-modal representation learning, advanced attention mechanisms, and label-efficient training strategies.

Several studies have directly targeted the unique challenges of RRSIS. Yuan et al. [46] develop the first dedicated RRSIS framework based on LAVT, introducing a language-guided cross-scale enhancement module that integrates deep and shallow features with linguistic cues to improve the segmentation of small and spatially dispersed objects. RMSIN [47] addresses multi-scale and orientation variability through intra- and cross-scale interaction modules combined with adaptive rotated convolution. CroBIM [48] explores bidirectional cross-modal interaction, employing context-aware prompt modulation for text encoding, language-guided feature aggregation for cross-scale dependencies, and a cascaded mutual-interaction decoder to achieve precise vision–language alignment. These studies highlight the importance of explicitly modeling multi-scale dependencies and cross-modal interactions to handle the inherent complexity of remote sensing imagery.

Complementing these task-specific frameworks, the adoption of large-scale vision–language foundation models has fundamentally advanced RRSIS. RSRefSeg [49] introduces a 1.2B-parameter architecture that transfers knowledge from CLIP and SAM via a novel AttnPrompter for coarse-to-fine segmentation. CADFormer [50] further enhances fine-grained cross-modal alignment through a semantic mutual guidance alignment module and a textual-enhanced cross-modal decoder, explicitly incorporating language semantics during decoding. LSCF [51] mitigates information loss through long-term semantic guidance combined with a ConvFormer backbone, integrating multiscale CoordConv and cross-modal attention modules to extract features across diverse receptive fields. Collectively, these works demonstrate a shift from task-specific networks toward foundation model–augmented solutions with improved generalization and scalability.

Given the dense and heterogeneous spatial distribution of objects in remote sensing imagery, modeling multi-scale interactions and spatial attention remains critical. IPFA-Net [52] introduces farthest point sampling–important points sampling to preserve geometric information during down-sampling while connecting features across scales and densities. LQVG [53] leverages sentence-level text embeddings as queries to retrieve visual representations from multi-scale features and contributes the high-resolution RSVG-HR dataset to benchmark language-guided segmentation on large-scale imagery. These methods underscore the necessity of spatially aware attention mechanisms and cross-scale feature aggregation to achieve robust RRSIS performance.

Because obtaining large-scale pixel-level annotations is costly in remote sensing, weakly supervised and semi-supervised approaches have gained increasing attention. CISM [54] exploits cross-image semantic mining using common semantic mining and non-common semantic contrastive loss functions, with a prototype interactive enhancement module to reinforce inter-image feature consistency. ASE-Net [55] implements an adversarial consistency training strategy with dual discriminators and a dynamic convolution-based bidirectional attention component for adaptive weight refinement. These approaches suggest that label-efficient learning constitutes a promising avenue for scaling RRSIS across diverse geographic regions.

Beyond discriminative modeling, generative paradigms, particularly diffusion models, are beginning to reshape RRSIS research. DMDC [56] adapts denoising diffusion probabilistic models to remote sensing super-resolution, incorporating detail complement mechanisms that significantly improve the reconstruction of small and dense targets. In parallel, the multimodal co-learning taxonomy [57] provides a principled framework to address challenges such as missing modalities, noisy inputs, and modality imbalance, thereby guiding the design of robust multimodal referring segmentation systems.

## 3 Methodology

### 3.1 Preliminaries: diffusion models

Diffusion probabilistic models rely on a parameterized Markov chain trained via variational inference to model data distributions. The key idea is to gradually transform data into noise (forward process) and learn its reverse (backward process), enabling sample generation from noise.

**Fig. 1:**
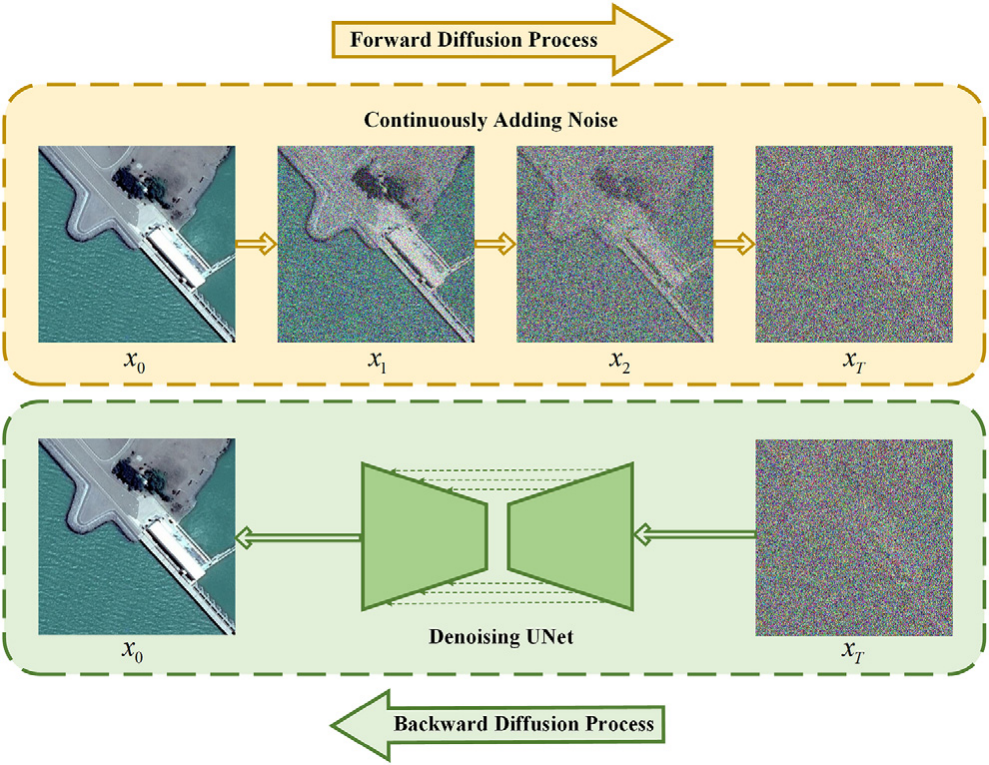
**Schematic illustration of diffusion probabilistic models.** The forward diffusion process (top) gradually adds noise to the original image. The backward diffusion process (bottom) learns to reverse this degradation through a denoising UNet, progressively recovering the original image from pure noise.

**Forward Process:** As shown in Fig. 1, starting from an image $x_{0}$, the forward process adds Gaussian noise over T steps:

(1) $$q\left(x_{t}|x_{t-1}\right)=N\left(x_{t};\sqrt{1-\beta_{t}}x_{t-1},\beta_{t}I\right),$$

where $\beta_{t}$ is a variance schedule. The full process is:

(2) $$q\left(x_{1:T}|x_{0}\right)=\prod_{t=1}^{T}q\left(x_{t}|x_{t-1}\right).$$

This allows direct sampling from any timestep t:

(3) $$q\left(x_{t}|x_{0}\right)=N\left(x_{t};\sqrt{{\bar{\alpha }}_{t}}x_{0},\left(1-{\bar{\alpha }}_{t}\right)I\right),$$

with $\alpha_{t}=1-\beta_{t}$, ${\bar{\alpha }}_{t}=\prod_{i=1}^{t}\alpha_{i}$.

**Backward Process:** The reverse process approximates $q(x_{t-1}|x_{t})$ using a neural network $p_{\theta }$:

(4) $$p_{\theta }\left(x_{t-1}|x_{t}\right)=N\left(x_{t-1};\mu_{\theta }\left(x_{t},t\right),\Sigma_{\theta }\left(x_{t},t\right)\right),$$

yielding the full generative process:

(5) $$p_{\theta }\left(x_{0:T}\right)=p\left(x_{T}\right)\prod_{t=1}^{T}p_{\theta }\left(x_{t-1}|x_{t}\right),$$

where $x_{T}∼N\left(0,I\right)$ and θ are parameters learned to minimize the variational bound:

(6) $$L\left(\theta \right)=\text{KL}\left(q\left(x_{0:T}\right)∥p_{\theta }\left(x_{0:T}\right)\right).$$

### 3.2 DiffRIS framework

The proposed DiffRIS framework is designed to fully leverage the capabilities of text-to-image diffusion models by transferring their pre-trained knowledge from large-scale vision-language datasets to remote sensing applications, specifically for RRSIS tasks. By seamlessly integrating high-level semantic representations with low-level visual attributes, DiffRIS achieves robust cross-modal semantic alignment, effectively mapping textual descriptions to their corresponding regions within remote sensing imagery. With the incorporation of a query mechanism and explicit cross-modal guidance, DiffRIS excels in capturing multi-scale, fine-grained targets in complex, heterogeneous environments. Moreover, it harnesses the global contextual information inherent in generative models, thereby enhancing semantic understanding and improving performance in challenging remote sensing scenarios.

**Fig. 2:**
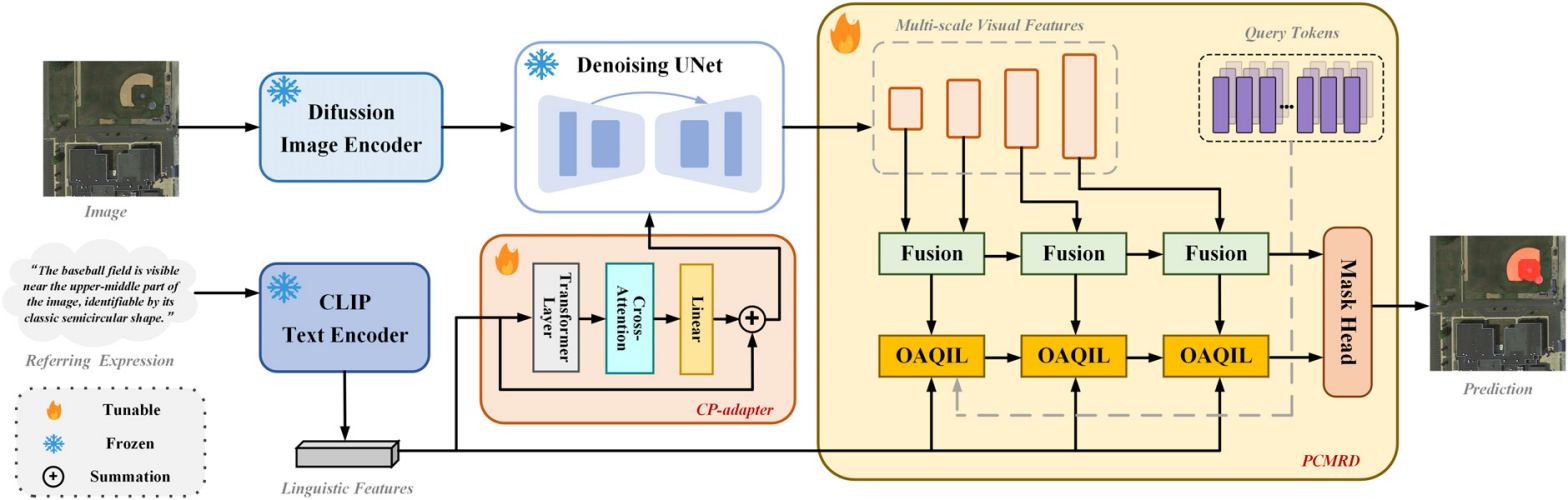
**Architecture of the proposed DiffRIS framework for referring remote sensing image segmentation.** The framework consists of three main components: (1) pre-trained CLIP text encoder, diffusion image encoder and denoising UNet that collaboratively extract multi-modal features, (2) a context perception adapter (CP-adapter) that refines linguistic features and bridges the domain gap, and (3) a progressive cross-modal reasoning decoder (PCMRD) that enables fine-grained segmentation through cross-modal interaction. During training, only the CP-adapter and PCMRD are optimized while all pre-trained components remain frozen to preserve their learned representations.

The overall architecture of the proposed DiffRIS is illustrated in Fig. 2. The input image $I\in R^{H\times W\times \;3}$ is initially transformed into a latent space representation $z\in R^{h\;\times w\times c}$ by a pre-trained diffusion image encoder (i.e., VQGAN [58]), where H and W are the height and width of the image, and h, w, and c represent the height, width, and channel dimensions of the latent space, respectively. Simultaneously, a referring language expression $E=\left\{e_{i}\right\},i\in \left\{0,\ldots ,N\right\}$, where N is the number of tokens, is encoded into linguistic features $L\in R^{l_{m}\times D_{l}}$ using a CLIP-based [59] text encoder. In this context, $l_{m}$ specifies the maximum length of the token sequence, while $D_{l}$ indicates the dimensionality of the linguistic feature space.

To mitigate the domain gap between the pre-training task and downstream tasks, we introduce a context perception adapter (CP-adapter) to obtain the refined linguistic features $L^{\prime }$. The refined linguistic features $L^{\prime }$ and the latent image representation z are then fed into a UNet-based denoising network to extract multi-scale visual features ${\left\{V_{i}\in R^{H_{i}\times W_{i}\times C_{i}}\right\}}_{i=1}^{4}$, where $H_{i}=H/2^{i+1}$ and $W_{i}=W/2^{i+1}$ represent the downsampled spatial resolutions derived from the original image dimensions H and W, and $C_{i}$ denotes the channel dimension of the i-th visual feature. Unlike traditional diffusion processes, no noise is added to the latent representation, and the denoising step is simplified to a single forward pass, streamlining feature extraction for downstream tasks.

Furthermore, we propose a progressive cross-modal reasoning decoder (PCMRD), which incorporates learnable query tokens Q to represent objects. By alternately querying linguistic features L and grouping multi-scale visual features ${\left\{V_{i}\right\}}_{i=1}^{4}$ into these tokens, the PCMRD enables object-aware interactions, thereby facilitating cross-modal reasoning to produce the final predictions.

During the training phase of the proposed DiffRIS framework, only the parameters of the proposed CP-adapter and PCMRD are updated, while all other parameters remain frozen. This strategy ensures that the pre-trained knowledge of the diffusion model and the vision-language encoders is preserved, allowing the framework to efficiently adapt to remote sensing scenarios.

### 3.3 Context perception adapter

**Fig. 3:**
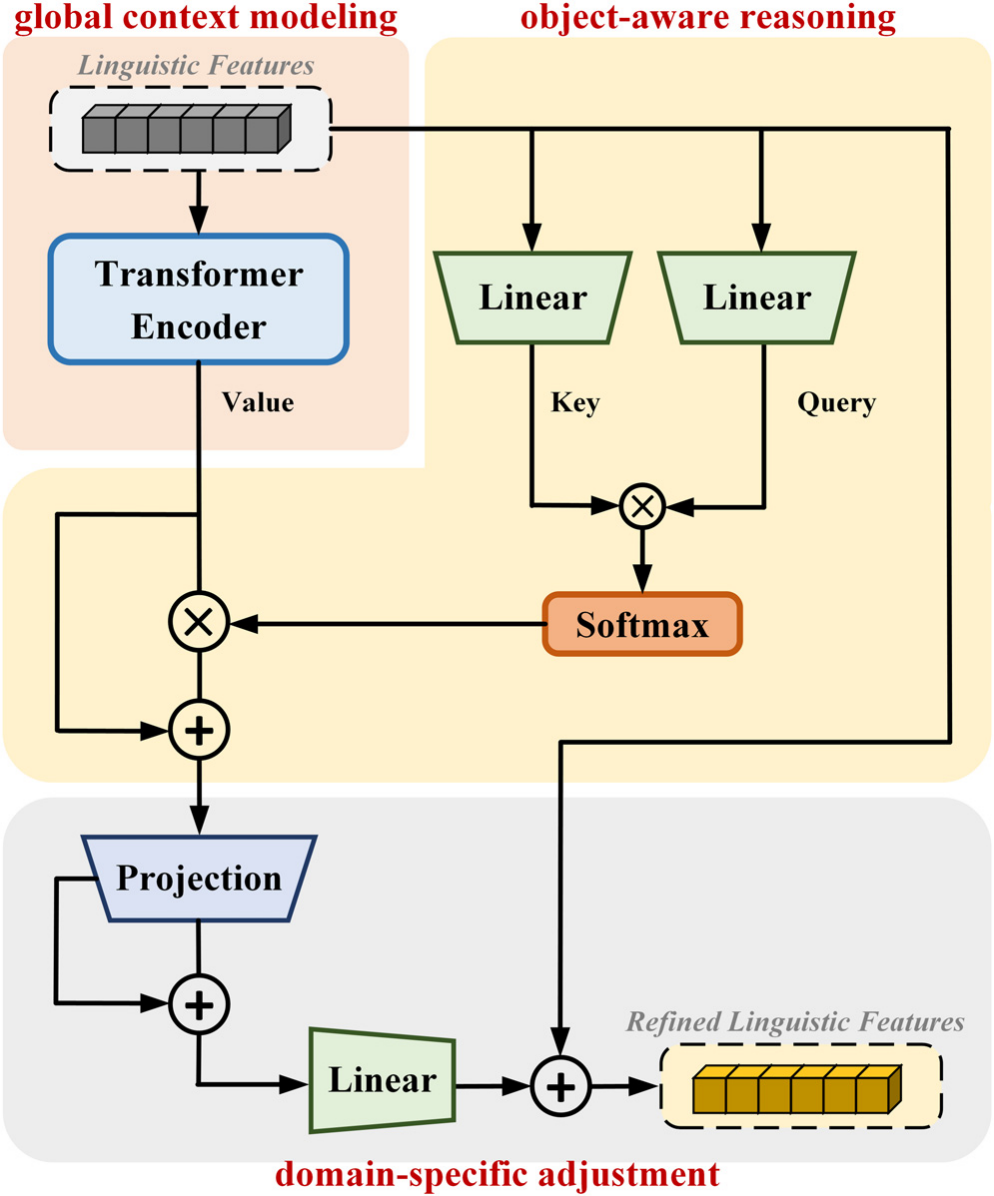
**Detailed architecture of the proposed CP-adapter.** The adapter comprises three key components: global context modeling via a Transformer encoder, object-aware reasoning through attention mechanisms, and domain-specific adjustment using low-rank adaptation. The refined linguistic features preserve pre-trained knowledge while adapting to remote sensing domain characteristics.

To effectively preserve the pre-trained knowledge of the text encoder and bridge the domain gap between pre-training tasks and downstream applications, we propose a context perception adapter (CP-adapter). The CP-adapter is specifically designed to refine linguistic features, enabling them to adapt to the domain-specific contextual requirements inherent in RRSIS tasks. This adapter utilizes a lightweight yet efficient architecture, incorporating three key components: global context modeling, object-aware reasoning, and domain-specific adjustment, ensuring optimal feature alignment and task adaptability (Fig. 3).

Initially, the Transformer encoder is employed to capture the global semantic relationships among linguistic tokens. The input linguistic features L are recursively processed through multiple layers of the Transformer encoder, resulting in preliminary context-aware representations H:

(7) $$H=\;\text{TransformerEncoder}\;\left(L\right)\in R^{l_{m}\times D_{l}},$$

In each layer of the Transformer encoder, global semantic dependencies are captured through a combination of multi-head self-attention (MHSA) mechanisms and feedforward neural networks.

Subsequently, task-relevant information is enhanced by iteratively applying attention mechanisms between the encoded features and the original input. Specifically, the query (Q), key (K), and value (V) are generated as follows:

(8) $$H_{v}=W_{v}H,L_{k}=W_{k}L,L_{q}=W_{q}L,$$

where $W_{v},W_{k},W_{q}\in R^{D_{l}\times D_{l}}$ are linear projection matrices. The attention score is then calculated as:

(9) $$\text{Attention}\left(L_{q},L_{k},H_{v}\right)=\text{softmax}\left(\frac{L_{q}L_{k}^{⊤}}{\sqrt{D_{l}}}\right)H_{v},$$

Subsequently, it is integrated with the input linguistic features L to generate enhanced feature representations $H_{\text{Enhanced}}$.

(10) $$H^{\prime }=\text{Attention}\left(L_{q},L_{k},H_{v}\right)+L,$$

(11) $$H_{\text{Enhanced}\;}=\text{Projection}\left(H^{\prime }\right)+H^{\prime },$$

where Projection(·) refers to a sequential block of two fully connected layers with a ReLU activation in between.

To further align the features with the requirements of downstream tasks, domain-specific adjustments are applied through a low-rank adaptation layer:

(12) $$H_{\text{LLM}\;}=W_{\text{LLM}\;}\cdot H_{\text{Enhanced}\;},$$

where $W_{\text{LLM}\;}$ represents the low-rank linear transformation matrix, with initial weights set to zero to prevent excessive perturbation during fine-tuning.

The CP-adapter generates the refined linguistic features $\hat{L}$ by weighted fusion of the original features L and the adjusted features $H_{\text{LLM}}$:

(13) $$\hat{L}=\alpha \cdot H_{\text{LLM}}+\left(1-\alpha \right)\cdot L,$$

where α is a learnable weight that balances the contribution of the adapted features and the original features.

Through the collaborative effect of the aforementioned steps, the CP-adapter not only preserves the rich semantic information encoded during the pre-training phase but also adapts to the specific requirements of downstream tasks. This effectively bridges the domain gap while maintaining high computational efficiency.

### 3.4 Progressive cross-modal reasoning decoder

Furthermore, we propose a progressive cross-modal reasoning decoder (PCMRD) that incorporates learnable query tokens for object representation. Through alternately performing query-language interaction and query-vision feature grouping across multiple scales, PCMRD enables fine-grained cross-modal reasoning, thereby enhancing the model’s capability in comprehending textual descriptions and localizing corresponding visual targets. PCMRD comprises three progressive decoding processes, each of which begins by integrating two levels of visual feature maps. These fused features are further combined with linguistic features and query tokens to serve as the input to the object-aware query interaction layer (OAQIL), which subsequently outputs the updated tokens.

**Fig. 4:**
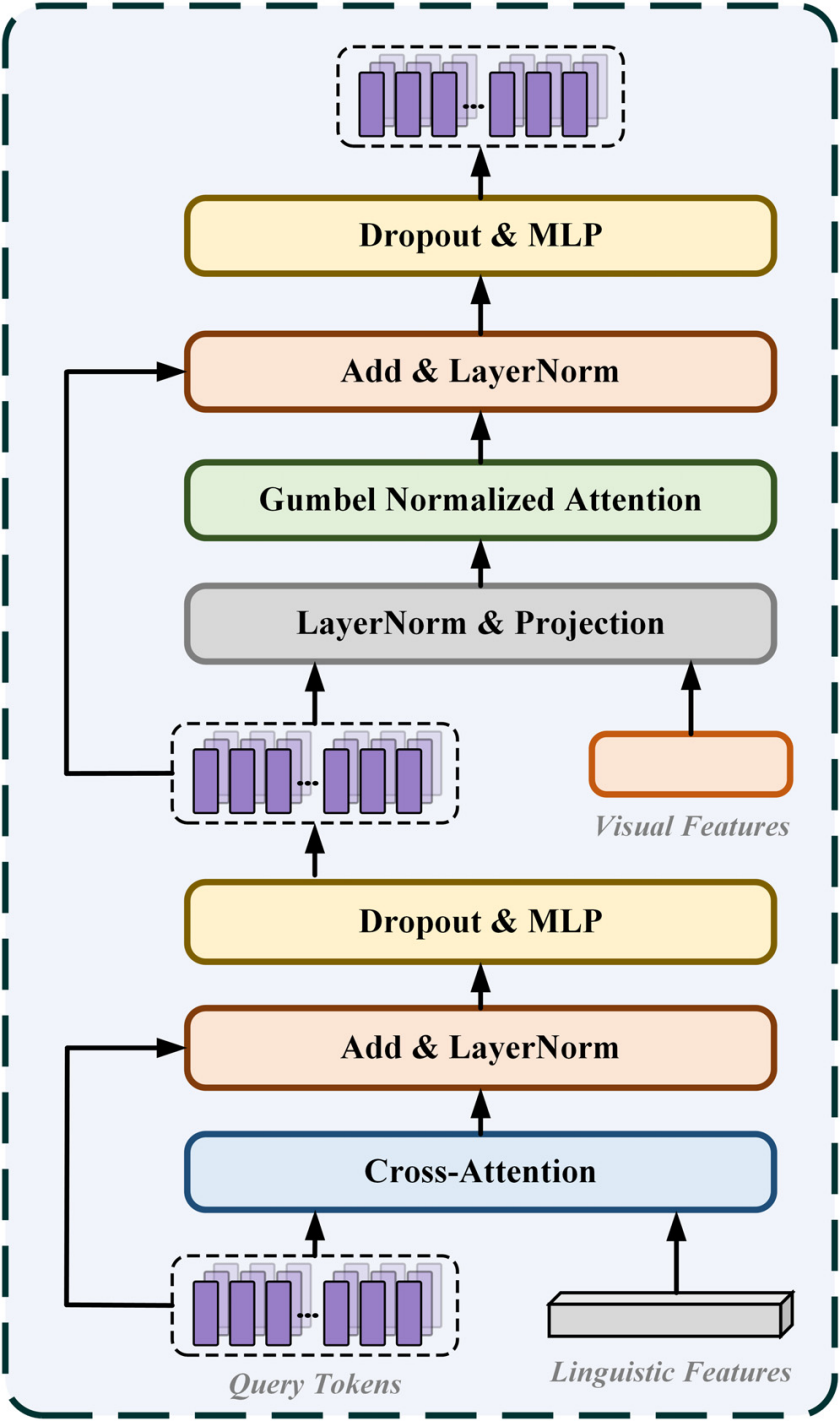
**Structure of the OAQIL within the PCMRD.** The OAQIL facilitates cross-modal reasoning through a series of attention mechanisms: cross-attention between query tokens and linguistic features, followed by Gumbel normalized attention for query-vision feature matching. Multi-layer perceptron (MLP) and normalization layers ensure effective feature integration.

As the core component of PCMRD, the OAQILs facilitate precise visual region localization through query text injection and adaptive query-vision feature matching, as illustrated in Fig. 4. For simplicity, we take the first decoding process as an example. Inspired by the success of query mechanisms in CV field, we introduce learnable query tokens $Q\in R^{M\times C_{q}}$ to encode object-centric representations, where M denotes the number of tokens and $C_{q}$ represents the token dimension. Next, the tokens Q are processed by OAQIL to capture the object-level information and update token features.

The input query tokens are first aligned with linguistic features through cross-attention, enabling each query token to focus on the relevant linguistic information required at the current layer. The output linguistic-enhanced tokens serve as a foundation for subsequent cross-modal reasoning and interaction:

(14) $$Q_{q}=W_{q}Q,L_{k}=W_{k}L,L_{v}=W_{v}L,$$

(15) $$Q_{l}=\left(\text{softmax}\left(\frac{Q_{q}{\left(L_{k}\right)}^{⊤}}{\sqrt{C_{q}}}\right)L_{v}\right)W_{c},$$

where $Q_{q}$, $L_{k}$ and $L_{v}$ represent the query, key, and value in the cross-attention mechanism, respectively, while $W_{c},W_{q}\in R^{C_{q}\times C_{q}}$ and $W_{k},W_{v}\in R^{D_{l}\times C_{q}}$ are learnable projection matrices. The generated linguistic-enhanced tokens $Q_{l}$ are subsequently fed into the Gumbel normalized attention mechanism to query and associate relevant visual features with the tokens.

Subsequently, the fused cross-scale visual features $V_{3,4}^{\text{fused}}\in R^{H_{3}\times W_{3}\times C_{1}^{f}}$and linguistic-enhanced query tokens $Q_{l}$ undergo cross-modal attention interaction facilitated by the designed Gumbel normalized attention mechanism.

$Q_{l}$ and $V_{3,4}^{\text{fused}}$ are first projected into a common feature space, and we calculate the similarities $S_{\text{pixel}\;}\in R^{M\times H_{3}W_{3}}$ between them:

(16) $$S_{\text{pixel}\;}=\text{norm}\left(W_{t}Q_{l}\right)\text{norm}{\left(\text{flatten}\left(W_{d}V_{3,4}^{\text{fused}}\right)\right)}^{⊤},$$

where $W_{t}\in R^{C_{q}\times C_{q}}$ and $W_{d}\in R^{C_{1}^{f}\times C_{q}}$ denote the learnable projection matrices. The flatten operation reshapes the feature $V_{3,4}^{\text{fused}}$ into the visual feature with $H_{3}W_{3}$ vectors, while norm refers to $L_{2}$ normalization.

Based on the similarities and the learnable Gumbel-softmax [60,61], the features in $V_{3,4}^{\text{fused}}$ are then hard-assigned to the tokens $Q_{l}$, resulting in the generation of updated tokens $Q_{u}$ through the designed OAQIL. Unlike standard softmax, which produces dense, probabilistic assignments, Gumbel-Softmax enables near-discrete, one-hot-like outputs through its temperature-controlled relaxation. This property is particularly advantageous for hard-assigning visual features to query tokens, as it mitigates the ambiguity of overlapping attention weights common in standard softmax. Furthermore, compared to sparse attention mechanisms, Gumbel-Softmax provides a principled way to learn sparse feature-to-token assignments dynamically, avoiding the need for heuristic sparsity constraints:

(17) $$S_{\text{gumbel}\;}=\text{softmax}\left(\left(S_{\text{pixel}\;}+G\right)/\tau \right),$$

(18) $$S_{\text{onehot}\;}=\text{onehot}\left(\text{argma}x_{N}\left(S_{\text{gumbel}\;}\right)\right),$$

(19) $$S_{\text{mask}\;}={\left(S_{\text{onehot}\;}\right)}^{⊤}-\text{sg}\left(S_{\text{gumbel}\;}\right)+S_{\text{gumbel}\;},$$

(20) $$Q_{u}=\text{MLP}\left(S_{\text{mask}}\cdot \text{flatten}\left(W_{d}V_{3,4}^{\text{fused}}\right)\right)+W_{t}Q_{l}$$

where $G\in R^{M\times H_{3}W_{3}}$ is sampled from the Gumbel (0,1) distribution, and sg denotes the stop gradient operator. The $\text{argma}x_{N}$ selects the token with the highest similarity for each visual feature, while the one-hot operation encodes these token indices into one-hot vectors $S_{\text{onehot}}\in R^{H_{3}W_{3}\times M}$. The resulting mask $S_{\text{mask}\;}\in R^{M\times H_{3}W_{3}}$ represents the assignment of features from $V_{3,4}^{\text{fused}}$ to $Q_{l}$.

The fused visual features $V_{3,4}^{\text{fused}}$ are obtained by upsampling $V_{4}$ to match $V_{3}$’s spatial resolution, concatenating them along the channel dimension, and projecting through a 1 × 1 convolution: $V_{3,4}^{\text{fused}}=\text{ReLU}\left(\text{BN}(\text{Con}v_{1\;\times \;1}\left[V_{3};V_{4}^{\text{up}}\right])\right)\in R^{H_{3}\times W_{3}\times C_{1}^{f}}$. This concatenation-projection strategy preserves complete multi-scale representations before fusion. Subsequent stages apply the same mechanism with $C_{2}^{f}$ and $C_{3}^{f}$.

Besides, the temperature coefficient τ is initialized to 1.0 and updated as a learnable parameter during training. To ensure numerical stability and effective gradient flow, τ is constrained to the range [0.1,5.0] via clamping after each update. This setup allows the model to dynamically balance discrete assignment behavior and gradient smoothness, and follows practices established in prior works [60,61].

### 3.5 Training objective

The training objective of DiffRIS is designed to address the inherent challenges in referring remote sensing image segmentation, particularly the severe class imbalance between target objects and background regions, as well as the need for effective multi-scale feature learning. Our loss function combines dice loss and sigmoid focal loss in a multi-scale supervision framework to ensure robust training convergence and accurate segmentation performance.

The total training loss integrates these complementary components as follows:

(21) $$L_{\text{total}\;}=L_{\text{dice}\;}\left(\hat{M},M\right)+L_{\text{focal}\;}\left(\hat{M},M\right)+\sum_{i=1}^{3}\lambda_{i}\cdot L_{\text{dice}\;}\left({\hat{M}}_{i},M_{i}\right)$$

where $\hat{M}$ and M denote the predicted and ground truth segmentation masks respectively, ${\hat{M}}_{i}$ represents the intermediate predictions from the ii i-th decoder layer, and $M_{i}$ is the corresponding ground truth mask appropriately resized to match the spatial resolution of each intermediate layer. The dice loss, which directly optimizes the overlap between predicted and ground truth regions, is formulated as:

(22) $$L_{\text{dice}\;}\left(\hat{M},M\right)=1-\frac{2\sum_{x,y}\sigma \left({\hat{M}}_{x,y}\right)\cdot M_{x,y}+\epsilon }{\sum_{x,y}\sigma \left({\hat{M}}_{x,y}\right)+\sum_{x,y}M_{x,y}+\epsilon }$$

where σ(·) denotes the sigmoid activation function applied to the model outputs, (x,y) indexes spatial positions, and ϵ=1 serves as a smoothing constant for numerical stability. This formulation provides balanced gradient signals even when target objects occupy small portions of the image, a common scenario in remote sensing applications.

The sigmoid focal loss component further addresses the imbalance between easy and hard examples:

(23) $$L_{\text{focal}\;}\left(\hat{M},M\right)=-\alpha_{t}{\left(1-p_{t}\right)}^{\gamma }\text{log}\left(p_{t}\right)$$

where $p_{t}=p\cdot M+\left(1-p\right)\cdot \left(1-M\right)$ with $p=\sigma (\hat{M})$ representing the predicted probability after sigmoid activation. Based on empirical validation, we set α=−1 (no class-specific weighting) and γ=0, effectively reducing this to a standard binary cross-entropy loss that complements the dice loss effectively in our framework.

The multi-scale supervision strategy employs intermediate predictions from the PCMRD decoder to enhance feature learning at different semantic levels. The supervision weights are set as $\lambda_{1}=0.001$, $\lambda_{2}=0.01$, and $\lambda_{3}=0.1$ for the three decoder stages, reflecting increasing importance for predictions at higher resolutions. When spatial dimensions of intermediate predictions differ from the ground truth, nearest-neighbor interpolation is applied to ensure proper alignment before loss computation. This multi-scale approach ensures that the model learns robust representations across different feature scales, which is particularly crucial for handling the diverse object sizes and complex spatial patterns characteristic of remote sensing imagery. During training, gradients are computed only for the CP-adapter and PCMRD parameters, while the pre-trained diffusion model components remain frozen to preserve their learned representations, enabling efficient adaptation to the RRSIS task while maintaining the rich semantic knowledge encoded in the pre-trained models.

## 4 Experiments

### 4.1 Experimental settings

#### 4.1.1 Datasets

**Fig. 5:**
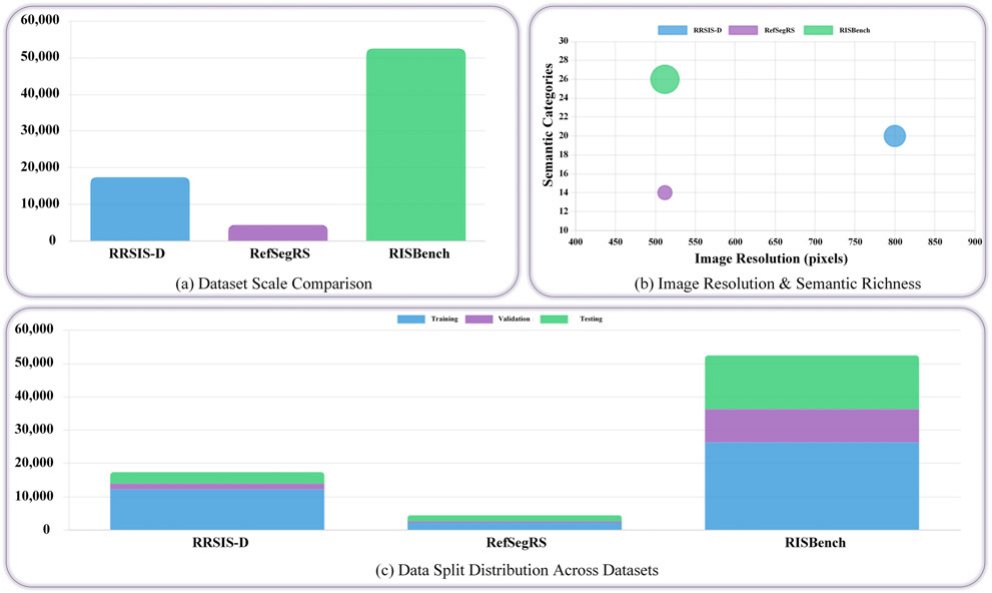
**Comparative statistical analysis and performance characteristics of RRSIS datasets**.

To evaluate the effectiveness of our algorithm, we conducted experiments on three public datasets: the RRSIS-D dataset [47], the RefSegRS dataset [46], and the RISBench dataset [48]. As illustrated in Fig. 5, these datasets exhibit significant variations in scale, resolution, and semantic complexity, providing comprehensive evaluation across different sample difficulty levels.

The RRSIS-D dataset features a diverse collection of 17,402 images, each paired with referring expressions and corresponding masks. It includes 20 semantic categories enriched by seven descriptive attributes, enabling detailed semantic context representation. The dataset is organized into training (12,181 triplets), validation (1740 triplets), and testing subsets (3481 triplets). Images are standardized at a resolution of 800 × 800 pixels, emphasizing large-scale and complex spatial scenes.

The RefSegRS dataset comprises 4420 image-language-label triplets across 285 distinct scenes. The data is divided into training (151 scenes, 2172 expressions), validation (31 scenes, 431 expressions), and testing subsets (103 scenes, 1817 expressions). All images are formatted to 512 × 512 pixels with a spatial resolution of 0.13 m, making it suitable for fine-grained RRSIS tasks.

The RISBench dataset comprises 52,472 triplets, significantly expanding the scale and diversity of referring image segmentation benchmarks. The dataset is split into training (26,300 triplets), validation (10,013 triplets), and testing (16,159 triplets) subsets. Images, uniformly sized at 512 × 512 pixels, span spatial resolutions from 0.1 to 30 m, catering to multi-scale segmentation challenges. Semantic labels are distributed across 26 unique classes, each annotated with 8 descriptive attributes, offering comprehensive semantic and contextual richness.

#### 4.1.2 Evaluation metrics

To evaluate the performance of our algorithm as well as other comparison methods, we employ several standard evaluation metrics. These include the overall Intersection-over-Union (oIoU), mean Intersection-over-Union (mIoU), and precision at different threshold values X ∈ {0.5,0.6,0.7,0.8,0.9} (Pr@X). oIoU is calculated as the ratio of the total intersection area to the total union area across all test samples. This metric emphasizes the segmentation performance on larger objects by aggregating the intersection and union areas before computing the ratio:

(24) $$\text{oIoU}=\frac{\sum_{t}I_{t}}{\sum_{t}U_{t}}$$

mIoU, on the other hand, is the average of the IoU scores computed individually for each test sample. This approach ensures that both small and large objects contribute equally to the final metric, providing a balanced evaluation of the model’s performance across varying object sizes:

(25) $$\text{mIoU}=\frac{1}{K}\sum_{t=1}^{K}\frac{I_{t}}{U_{t}}$$

where t indexes each image-language-label triplet in the dataset, K denotes the total number of samples, and $I_{t}$ and $U_{t}$represent the intersection and union areas between the predicted segmentation mask and the corresponding ground truth annotation for the t-th sample.

Pr@X measures the proportion of correctly segmented objects that achieve an IoU greater than or equal to the threshold X. This metric provides insight into the model’s ability to produce high-quality segmentations under varying strictness levels.

#### 4.1.3 Training details

The model was implemented and trained using the PyTorch framework, leveraging a hardware setup of eight NVIDIA A800 GPUs, each equipped with 80GB of memory. Besides, the model was optimized using the AdamW optimizer with a learning rate of $3\;\times 10^{-5}$, a weight decay of 0.01, and a batch size of 32. The training process is conducted over 40 epochs to ensure sufficient optimization and convergence. During both training and evaluation, input images were resized to a resolution of 512 × 512 pixels. Notably, no data augmentation or post-processing techniques were employed throughout the entire pipeline.

### 4.2 Performance analysis

To evaluate the effectiveness of the proposed approach, we conducted comparative experiments using a selection of state-of-the-art frameworks, including ResNet-LSTM [7,15,39,41,62,63], ResNet-CLIP [43,64,65], Swin-Bert [40,46,47,[66], [67], [68], [69]]; S.-A. [13], and Stable Diffusion-CLIP [28,70], as benchmarks.

#### 4.2.1 Quantitative results

Table 1, Table 2, Table 3 present a comprehensive comparison of RIS performance across the RRSIS-D, RefSegRS, and RisBench datasets. Specifically, the tables report precision at various threshold values (Pr@X), as well as oIoU and mIoU metrics, for both the validation and test sets. The optimal and sub-optimal performances are distinctly highlighted in red and blue, respectively.

**Table 1:** **Comparison with state-of-the-art methods on the RefSegRS dataset**.

Optimal and sub-optimal performance in each metric are marked by  and .

**Table 2:** **Comparison with state-of-the-art methods on the RefSegRS dataset**.

Optimal and sub-optimal performance in each metric are marked by  and *.*

**Table 3:** **Comparison with state-of-the-art methods on the RISBench dataset**.

Optimal and sub-optimal performance in each metric are marked by  and *.*

On the RRSIS-D dataset, the proposed DiffRIS framework demonstrates outstanding performance, consistently outperforming all state-of-the-art methods across most metrics. DiffRIS achieves Pr@0.5 values of 73.45 (Val) and 71.79 (Test), surpassing the next best competitor, CARIS, by 1.84% and 0.29%, respectively. This advantage extends across higher thresholds (Pr@0.6 to Pr@0.9), underscoring its robustness in maintaining high precision under stricter conditions. Additionally, in terms of mIoU, DiffRIS achieves 63.64 (Val) and 62.19 (Test), further surpassing CARIS by 0.76% and 0.07%, respectively. These results underline its ability to handle diverse and complex segmentation tasks effectively.

On the RefSegRS dataset, DiffRIS achieves remarkable superiority, consistently securing optimal results across all metrics. Notably, it achieves Pr@0.5 values of 94.66 (Val) and 74.57 (Test), outperforming the second-best method, VPD, by a substantial margin of 7.89% and 5.33%, respectively. This dominance extends to higher thresholds (Pr@0.7 and Pr@0.8), demonstrating the framework’s precision and resilience in challenging scenarios. Furthermore, DiffRIS records the highest mIoU scores of 78.28 (Val) and 62.38 (Test), surpassing VPD by 10.28% and 5.08%, respectively. Its oIoU score of 85.63 (Val) is also the highest among all methods, emphasizing its robustness in handling complex spatial contexts.

On the RISBench dataset, DiffRIS continues to exhibit exceptional performance, surpassing all existing methods. It achieves Pr@0.5 values of 73.99 (Val) and 74.50 (Test), outperforming CARIS by 0.53% and 0.56%, respectively. This trend is consistently observed at higher thresholds, such as Pr@0.8 and Pr@0.9, indicating DiffRIS’s capability to precisely segment objects under stringent overlap requirements. In terms of segmentation quality, DiffRIS achieves the highest mIoU scores of 66.32 (Val) and 67.04 (Test), surpassing CARIS by 1.92% and 1.25%, respectively. The competitive oIoU results further validate its global consistency and segmentation effectiveness.

Across all three datasets, DiffRIS exhibits unparalleled performance, attributed to its innovative integration of Stable Diffusion and CLIP models. This approach effectively enhances cross-modal alignment and enables multi-scale feature representation, resulting in robust, precise, and generalizable segmentation capabilities.

#### 4.2.2 Qualitative results

**Fig. 6:**
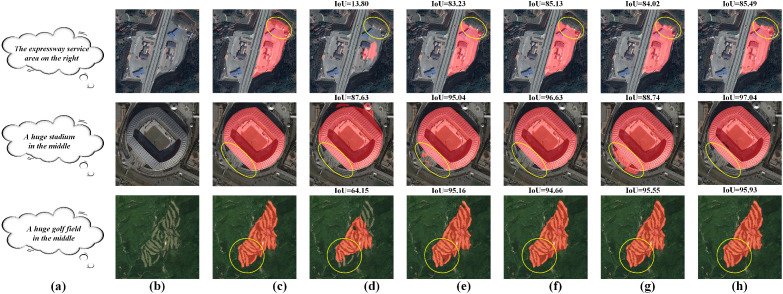
**Segmentation results of DiffRIS and baselines on the RRSIS-D test set with IoU scores.** (a) Query Expressions; (b) Input Images; (c) Ground Truths; (d) EVP; (e) LAVT; (f) RMSIN; (g) CARIS; (h) DiffRIS. Yellow circles highlight regions where DiffRIS outperforms baselines.

**Fig. 7:**
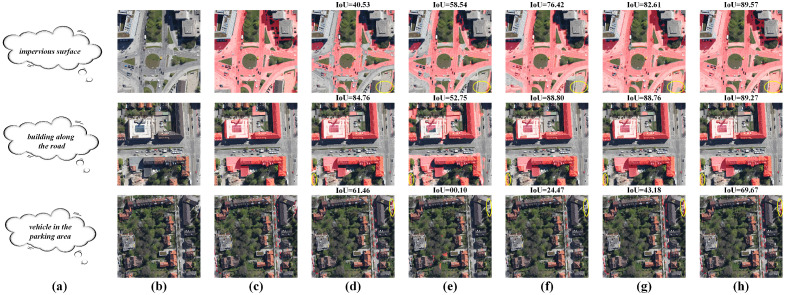
**Segmentation results of DiffRIS and baselines on the RefSegRS test set with IoU scores.** (a) Query Expressions; (b) Input Images; (c) Ground Truths; (d) LGCE; (e) EVP; (f) VPD; (g) ETRIS; (h) DiffRIS. Yellow circles highlight regions where DiffRIS outperforms baselines.

**Fig. 8:**
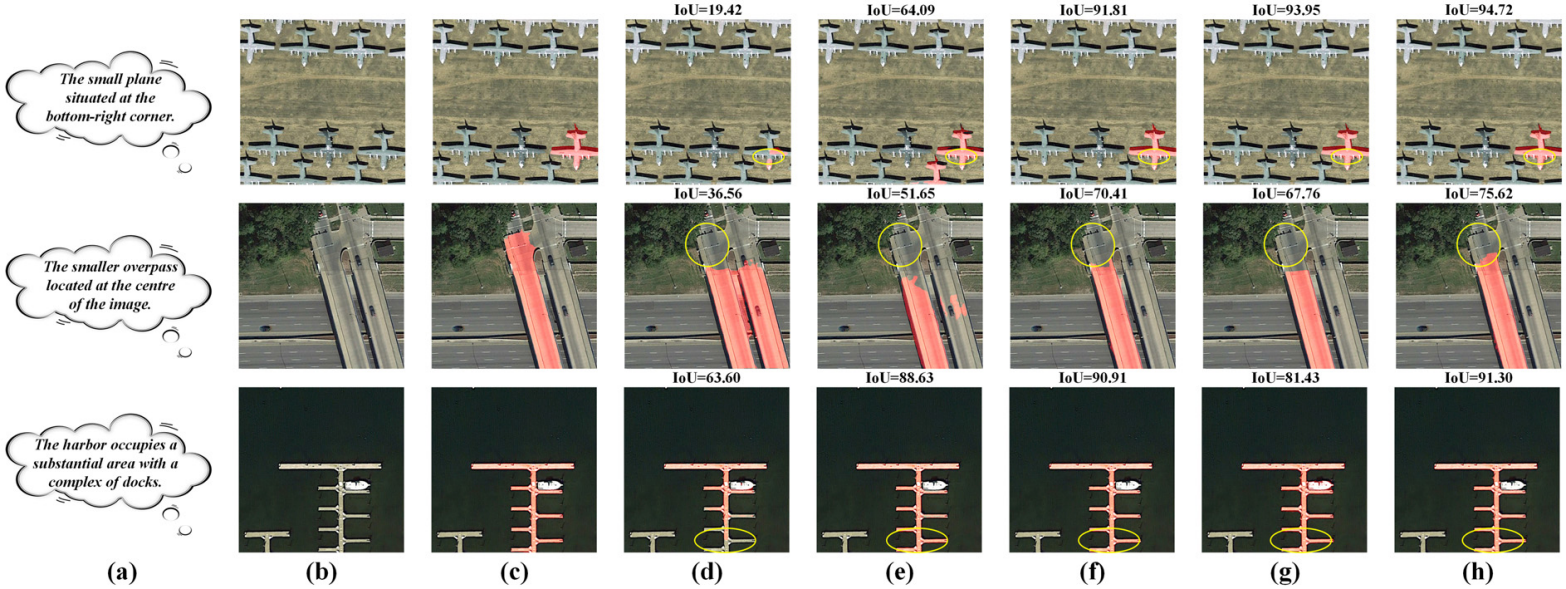
**Segmentation results of DiffRIS and baselines on the RisBench test set with IoU scores.** (a) Query Expressions; (b) Input Images; (c) Ground Truths; (d) EVP; (e) LAVT; (f) VPD; (g) CrossVLT; (h) DiffRIS. Yellow circles highlight regions where DiffRIS outperforms baselines.

Fig. 6, Fig. 7, Fig. 8 present the visual results of the RRSIS-D, RefSegRS, and RISBench datasets, supplemented by the corresponding IoU scores for each segmentation output. These visual comparisons highlight the superior performance of the proposed DiffRIS framework, which consistently produces more accurate and cohesive segmentation results across diverse and challenging scenarios. Notably, DiffRIS excels in handling intricate cases involving small or overlapping objects, occlusions, and ambiguous boundaries. In contrast to competing methods, DiffRIS demonstrates a remarkable ability to preserve fine-grained details while maintaining global consistency. Furthermore, it effectively mitigates common segmentation artifacts such as under-segmentation and over-segmentation, leading to significantly improved visual coherence. Overall, the visual evidence underscores DiffRIS’s ability to accurately model complex spatial and textual relationships, highlighting its efficacy and generalizability across a wide range of datasets and RRSIS applications.

### 4.3 Ablation studies

In this section, we conduct extensive ablation experiments on the RRSIS-D test set to demonstrate the effectiveness of each component within our DiffRIS.

#### 4.3.1 Effects on different pre-trained weights

In this study, we investigate the impact of pre-trained weights from various versions of the Stable Diffusion model on the performance of our DiffRIS framework. Given that DiffRIS is built upon pre-trained text-to-image diffusion models, understanding the influence of different pre-trained weights on model performance is of paramount importance. Specifically, we compare the performance of DiffRIS using weights from four different versions of the Stable Diffusion model: SD-1-1, SD-1-2, SD-1-4, and SD-1-5. We exclude SD-1-3 due to its training iteration count being 30K fewer than SD-1-4. The primary distinction among these versions lies in the number of pre-training iterations conducted on 512 × 512 resolution images, with higher versions benefiting from more extensive training.

**Fig. 9:**
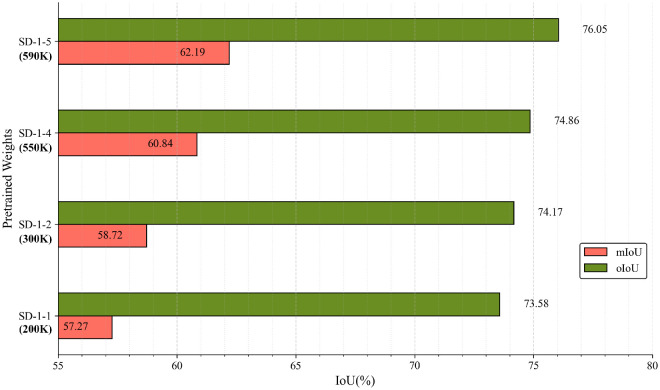
**Performance comparison of DiffRIS using different pre-trained weights from Stable Diffusion models on the RRSIS-D test set.** The metrics mIoU and oIoU are plotted for four versions of Stable Diffusion (SD-1-1, SD-1-2, SD-1-4, SD-1-5), demonstrating a consistent improvement in performance with increasing pre-training iterations.

As depicted in Fig. 9, a clear and consistent improvement in both mIoU and oIoU metrics is observed as the number of pre-training iterations increases. Notably, we observe a steady upward trend in performance across the various releases of the Stable Diffusion model, from SD-1-1 to SD-1-5. The mIoU value for SD-1-5 reaches 62.19%, markedly outperforming SD-1-1, which attains only 57.27%. This represents a significant enhancement of 4.92% in mIoU. Similarly, the oIoU score for SD-1-5 rises to 76.05%, surpassing the 73.58% of SD-1-1 by 2.47%. These results underscore a positive correlation between the number of pre-training iterations and the model’s ability to capture finer details, thereby producing more accurate and robust results on the RRSIS task. We attribute the performance gains from SD-1-5 to both broader concept coverage enabled by large-scale pre-training and improved latent space regularization, which jointly enhance the model’s ability to align diverse referring expressions with visual semantics in remote sensing imagery.

#### 4.3.2 Effects on the CP-adapter

To investigate the efficacy of the CP-adapter, we conduct an ablation study on the RRSIS-D test set within the DiffRIS framework, progressively adding key components of the text adapter. As shown in Table 4, when the CP-adapter is removed, DiffRIS achieves the lowest segmentation performance, with a mIoU of 75.52% and an oIoU of 61.43%. These results underscore the critical role of the CP-adapter in RRSIS tasks, as its absence significantly impairs the model’s ability to preserve contextual information and align features effectively.

**Table 4:** **Ablation analysis of CP-adapter with different components**.

	Method	mIoU	oIoU
1	w/o CP-adapter	75.52	61.43
2	1 + global context modeling	75.43	61.49
3	2 + object-aware reasoning	75.88	61.82
4	3 + domain-specific adjustment	**76.05**	**62.19**

The best performance is shown in bold.

Subsequently, we incrementally reintroduce the three core components of the CP-adapter: global context modeling, object-aware reasoning, and domain-specific adjustment. The introduction of global context modeling does not yield a significant performance boost, primarily due to its limited capacity to address the nuanced, domain-specific challenges inherent in remote sensing tasks. However, as object-aware reasoning is added, the oIoU improves to 61.82%, and with the subsequent inclusion of domain-specific adjustment, the oIoU further increases to 62.19%. These results demonstrate the importance of each component, where global context modeling lays the foundation for broader contextual understanding, while object-aware reasoning and domain-specific adjustment are pivotal in refining the model’s RIS accuracy and domain adaptation capabilities.

Overall, the complete CP-adapter effectively bridges the domain gap between pre-training tasks and downstream remote sensing applications, significantly enhancing RRSIS performance. The CP-adapter refines linguistic features to adapt them to the specific demands of RRSIS tasks, ensuring optimal feature alignment and task adaptability. These findings highlight the indispensable role of the CP-adapter in improving the model’s RIS performance through its lightweight yet efficient architecture.

#### 4.3.3 Effects on the PCMRD

We first compare PCMRD with three other decoders: LMP, OAD, and CAMD, and the results are shown in Table 5. It is clear that PCMRD achieves the highest precision at multiple thresholds (Pr@0.5, Pr@0.7, and Pr@0.9), significantly outperforming the competing methods. This superior performance is attributed to the novel progressive cross-modal reasoning mechanism in PCMRD, which facilitates fine-grained interactions between visual and linguistic features. Unlike the decoders in LMP and OAD, which do not leverage cross-modal feature interactions, PCMRD integrates dynamic query-text and query-vision interactions, enabling more accurate object localization and segmentation. Furthermore, while CAMD captures valuable visual features, it neglects multi-scale information, which is crucial for handling objects of varying sizes and complex scenarios. In contrast, PCMRD excels in this aspect by employing multi-scale feature fusion, leading to enhanced performance in both global and local context understanding.

**Table 5:** **Ablation studies on decoders in DiffRIS**.

Decoder	Pr@0.5	Pr@0.7	Pr@0.9	mIoU	oIoU
LMP [40]	63.82	48.65	20.73	75.98	56.54
OAD [47]	65.99	49.83	21.52	75.62	58.31
CAMD (S.-A. [13])	71.32	52.17	23.51	**76.41**	61.86
PCMRD (ours)	**71.79**	**54.64**	**26.46**	76.05	**62.19**

The best performance is shown in bold.

Moreover, the ablation study in Table 6 highlights the contribution of each component in the PCMRD. The baseline is defined as the model that directly extracts visual features and predicts the segmentation results using dynamic convolutions from the linguistic features. In the baseline, introducing multi-scale fusion improves performance by capturing both global and local contextual information, resulting in an increase of mIoU to 74.29% and oIoU to 56.37%. Further, adding query tokens refines object-level understanding, leading to a further improvement in RRSIS performance. Finally, the use of hard assignment with learnable Gumbel Softmax refines token grouping, achieving the best performance with an mIoU of 76.05% and oIoU of 62.19%. These results demonstrate the effectiveness of each component of PCMRD in enhancing segmentation accuracy and object localization.

**Table 6:** **Ablation analysis of PCMRD with different components**.

	Method	mIoU	oIoU
1	Baseline	72.18	53.62
2	1 + multi-scale fusion	74.29	56.37
3	2 + query tokens	75.46	59.81
4	3 + hard assignment	**76.05**	**62.19**

The best performance is shown in bold.

## 5 Discussion

### 5.1 Computational efficiency

The computational efficiency of DiffRIS presents a compelling advantage over existing state-of-the-art methods, as demonstrated through comprehensive comparisons with RSRefSeg. As shown in Table 7, DiffRIS achieves substantial improvements across all key efficiency metrics while maintaining superior segmentation performance. The parameter count reduction of 21.3% (from 1226.71M to 966.03M parameters) demonstrates the effectiveness of our parameter-efficient design strategy, where only the CP-adapter and PCMRD components require training while preserving the rich representations learned by pre-trained diffusion models. More significantly, DiffRIS reduces memory consumption by 62.4% (from 9.82GB to 3.69GB), making it more accessible for deployment on resource-constrained hardware configurations commonly encountered in remote sensing applications. The inference time improvement is particularly noteworthy, with a 61.1% reduction (from 0.18s to 0.07s per image) that translates to a 157% increase in processing throughput (from 5.56 to 14.3 FPS). These efficiency gains stem from our architectural design choices, including the simplified single forward pass through the diffusion encoder (bypassing the iterative denoising process), the lightweight adapter mechanisms, and the streamlined cross-modal reasoning decoder. This computational advantage is crucial for practical remote sensing applications where large-scale imagery processing and real-time analysis capabilities are essential.

**Table 7:** **Comparative analysis of computational requirements**.

Computational efficiency	RSRefSeg	DiffRIS (Ours)
Parameters (M)	1226.71	**966.03**
		(↓21.3%)
Memory (GB)	9.82	**3.69**
		(↓62.4%)
Inference time (s/img)	0.18	**0.07**
		(↓61.1%)
FPS	5.56	**14.3**
		(↑157%)

Bold values indicate the best results.

### 5.2 Attention visualization

**Fig. 10:**
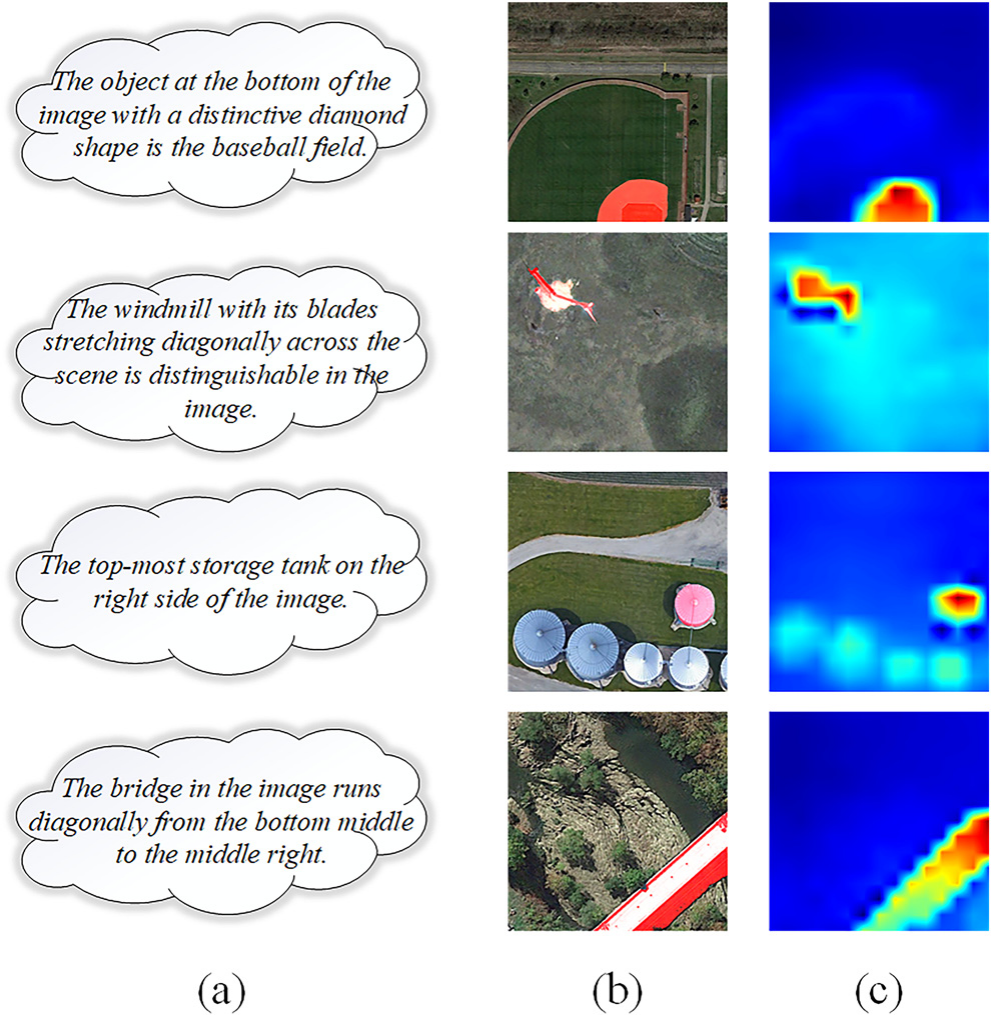
**Visualization of attention maps.** (a) Input query expressions; (b) Predicted segmentation masks; (c) Corresponding attention maps.

As shown in the Fig. 10, the attention visualization analysis provides compelling evidence for DiffRIS’s ability to establish precise semantic alignment between textual descriptions and visual regions in remote sensing imagery. Through comprehensive examination of attention heatmaps generated by our PCMRD, we observe that the activated regions closely correspond to the ground truth segmentation masks, demonstrating the model’s capacity to accurately interpret and localize textual referring expressions. The attention patterns reveal that DiffRIS effectively captures both coarse-grained semantic concepts and fine-grained spatial details, with attention weights concentrating on target objects while appropriately suppressing background regions. Particularly noteworthy is the model’s ability to handle complex spatial relationships and multi-scale objects, where attention mechanisms successfully focus on relevant object parts even when targets exhibit significant scale variations or are embedded within cluttered backgrounds typical of aerial imagery. The progressive nature of our cross-modal reasoning decoder enables iterative refinement of attention maps across different resolution levels, resulting in increasingly precise localization as information flows through the decoder stages. These visualization results validate our hypothesis that pre-trained diffusion models provide robust cross-modal alignment capabilities, while our proposed adapter and decoder components successfully bridge the domain gap between natural image understanding and remote sensing applications.

### 5.3 Robustness and generalization

**Fig. 11:**
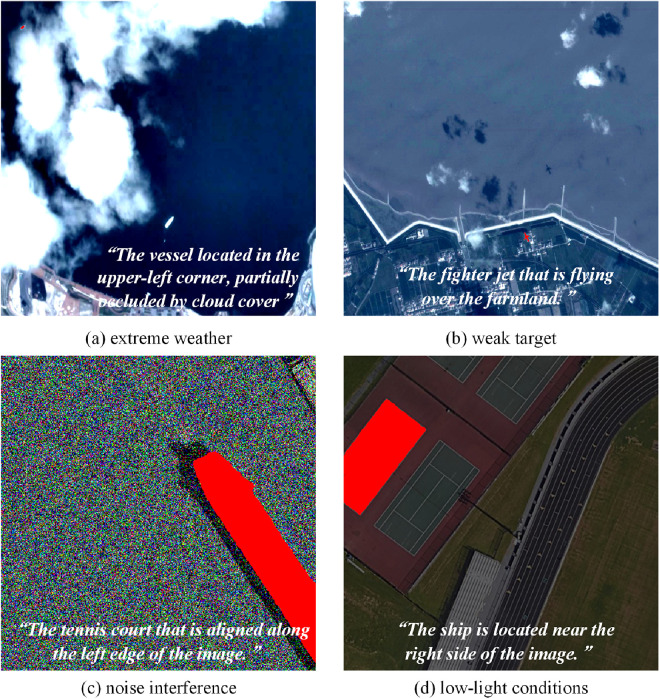
**Qualitative results under challenging real-world conditions.** Our DiffRIS demonstrates robust performance across diverse challenging scenarios: (a) extreme weather with heavy cloud cover, (b) weak target detection in complex backgrounds, (c) severe noise interference, and (d) low-light environments. Red regions indicate the predicted segmentation masks.

To evaluate the generalization capabilities and robustness of DiffRIS beyond the confines of standard benchmark datasets, as shown in Fig. 11, we conducted comprehensive experiments on real-world scenarios that present significant challenges for referring image segmentation systems. These practical evaluations demonstrate DiffRIS’s ability to maintain accurate performance when deployed in actual remote sensing applications where controlled laboratory conditions do not apply. Under extreme weather conditions, as illustrated in the vessel detection scenario with heavy cloud cover, DiffRIS successfully localizes and segments the target vessel despite significant occlusion and atmospheric interference that severely degrades visual clarity. The model’s ability to identify “the vessel located in the upper-left corner, partially occluded by cloud cover” demonstrates its resilience to meteorological variations commonly encountered in satellite and aerial imagery acquisition. The weak target detection experiment reveals DiffRIS’s exceptional capability in handling small-scale objects within expansive geographical contexts. The successful segmentation of “the fighter jet that is flying over the farmland” showcases the model’s proficiency in identifying targets that occupy minimal pixel regions relative to the overall image dimensions—a critical requirement for military and surveillance applications where target-to-background ratios are extremely low. This performance validates the effectiveness of our multi-scale feature processing and cross-modal reasoning mechanisms in preserving fine-grained spatial information across different resolution levels. Under severe noise interference conditions, where traditional computer vision algorithms typically fail due to signal degradation, DiffRIS maintains robust performance in segmenting “the tennis court that is aligned along the left edge of the image” despite substantial visual corruption. This resilience stems from the model’s ability to leverage textual semantic guidance to compensate for degraded visual information, effectively utilizing cross-modal complementarity. Finally, in low-light environments where reduced illumination and poor contrast conditions challenge conventional segmentation approaches, DiffRIS successfully identifies “the ship is located near the right side of the image,” demonstrating its capacity to operate effectively across varying lighting conditions encountered in different temporal and geographical contexts. These real-world evaluations collectively establish that DiffRIS transcends the limitations of dataset-specific performance metrics, providing genuine practical value for operational remote sensing applications where environmental variability and data quality uncertainties are inherent challenges. The model’s consistent performance across these diverse challenging scenarios validates its robustness and confirms its readiness for deployment in real-world remote sensing systems.

More detailed experimental results and analysis are provided in Appendices A and B.

## 6 Conclusion

In this work, we present DiffRIS, a diffusion-powered framework for Referring Remote Sensing Image Segmentation (RRSIS). We leverage frozen latent features from pre-trained diffusion models as visual representations and introduce a lightweight CP-adapter for text-image alignment, along with a Progressive Cross-Modal Reasoning Decoder (PCMRD) for semantic fusion. Experimental results on three RRSIS benchmarks demonstrate the superiority and robustness of DiffRIS over prior approaches across multiple metrics and backbones.

Despite these improvements, we acknowledge that the underlying diffusion-based encoder introduces non-trivial computational costs during inference, particularly in terms of GPU memory consumption. While our use of frozen backbones and parameter-efficient adapters ensures competitive runtime, deploying DiffRIS on resource-constrained platforms remains challenging. Future work may address this by exploring lightweight alternatives such as knowledge distillation, model quantization, or compact latent-space diffusion variants.

In addition, the framework may benefit from alternative diffusion backbones, such as DiT or VAR, which provide stronger global context modeling or autoregressive reasoning, albeit with potential trade-offs in computational efficiency and domain adaptability. We leave the exploration of these directions as promising avenues for future RRSIS research.

## CRediT authorship contribution statement

**Zhe Dong:** Writing – review & editing, Writing – original draft, Visualization, Validation, Supervision, Software, Resources, Project administration, Methodology, Investigation, Formal analysis, Data curation, Conceptualization. **Yu-Zhe Sun:** Writing – review & editing, Validation, Conceptualization. **Tian-Zhu Liu:** Validation, Funding acquisition. **Yan-Feng Gu:** Validation, Project administration, Funding acquisition, Conceptualization.

## Declaration of competing interest

The authors declare that they have no conflicts of interest in this work.

## Figures and Tables

**Fig. 12:**
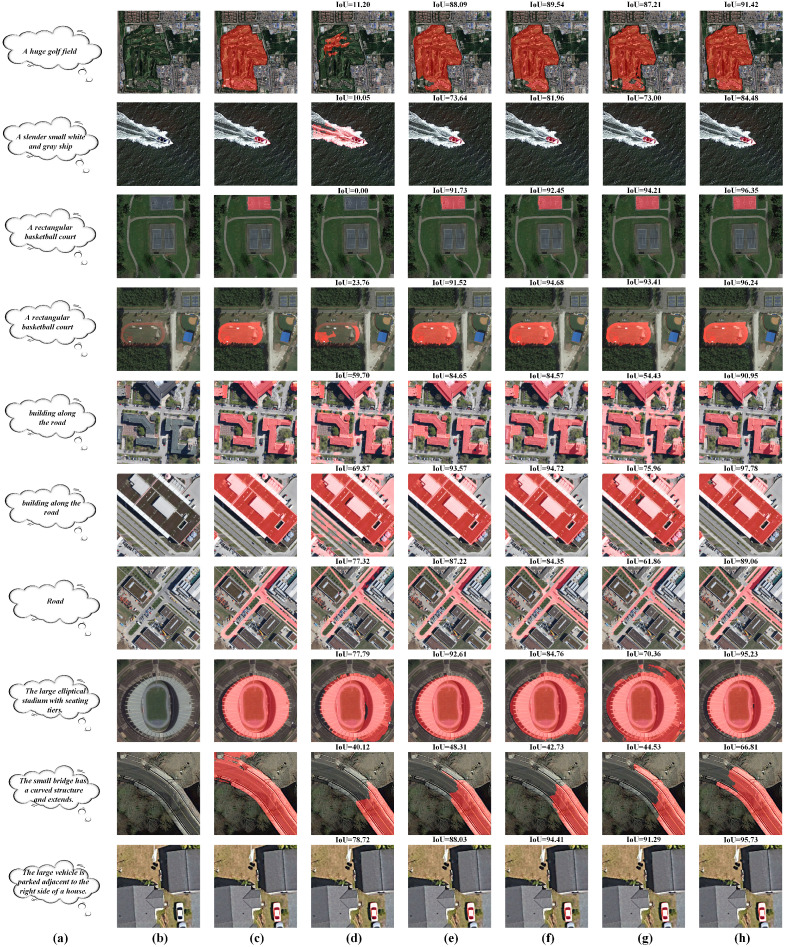
**Additional segmentation examples across RRSIS-D, RefSegRS, and RISBench datasets.** Each row shows: (a) Query Expressions; (b) Input Images; (c) Ground Truths; (d) EVP; (e) LAVT; (f) RMSIN; (g) VPD; (h) DiffRIS. These supplementary cases demonstrate consistent performance across diverse object categories and spatial configurations.

**Fig. 13:**
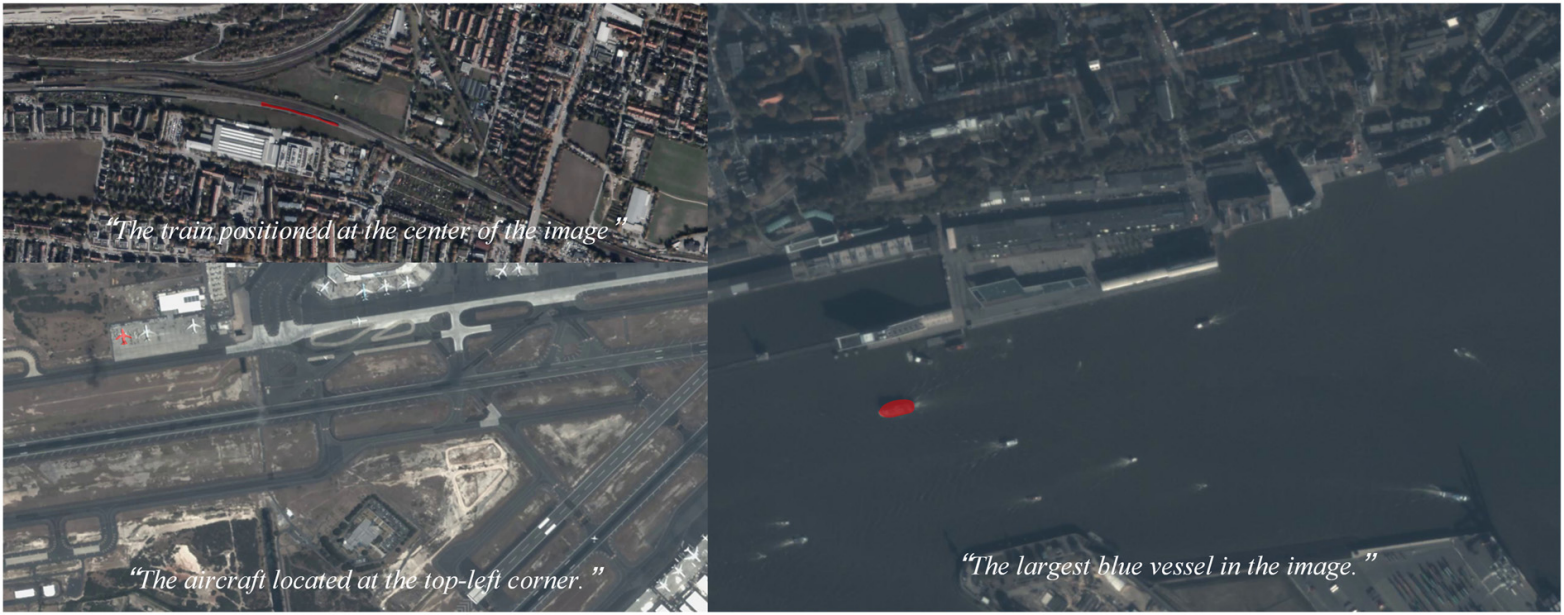
**Segmentation results on real-world large-scale Jilin-1 imagery, demonstrating DiffRIS’s capability to localize and segment small targets under complex operational conditions**.

## Appendix A. Additional segmentation results

To provide a more comprehensive performance evaluation, we present supplementary segmentation examples across the RRSIS-D, RefSegRS, and RISBench datasets. These additional visualizations complement the representative cases in Fig. 6, Fig. 7, Fig. 8 and demonstrate DiffRIS’s consistent performance across diverse object categories, scales, and spatial contexts.

Fig. 12 illustrates additional segmentation results spanning various semantic categories including buildings, roads, sports facilities, and transportation infrastructure. Each example displays the referring expression, input image, ground truth mask, the predictions from DiffRIS and the compared baseline methods, along with their corresponding IoU scores. The results demonstrate robust segmentation quality across different spatial resolutions and geographical contexts, effectively handling objects of varying scales, orientations, and complexity levels.

## Appendix B. Segmentation results on real-world large-scale imagery

To validate DiffRIS’s practical applicability, we present segmentation results on real-world large-scale imagery from Jilin-1 03 satellites. These images feature high spatial resolution (0.92 m) and large dimensions ranging from 2152 × 1080 to 2800 × 2000 pixels, presenting realistic challenges including varying illumination, atmospheric interference, and complex backgrounds.

Fig. 13 illustrates representative segmentation results on three critical object categories—ships, aircraft, and trains—which are of particular importance for transportation monitoring and defense applications. Each example displays the referring expression, input large-scale image, and DiffRIS prediction. The results demonstrate DiffRIS’s capability to accurately localize and segment these targets despite their small relative sizes within expansive scenes and challenging environmental conditions. The model successfully handles scale variations, orientation differences, and cluttered backgrounds while maintaining precise boundary delineation.
